# Decoding Yield Variability in Hungarian Vetch: A Biplot-Based Analysis Across Two Regions

**DOI:** 10.3390/plants14172669

**Published:** 2025-08-27

**Authors:** Emine Serap Kızıl Aydemir, Ali Devlet, Kağan Kökten, Abdulbaki Bilgiç

**Affiliations:** 1 Department of Field Crops, College of Agriculture and Natural Sciences, Seyh Edebali University, Gulumbe 11230, Bilecik, Türkiye; ali.devlet@bilecik.edu.tr; 2 Department of Animal and Rangeland Sciences, Oregon State University, Corvallis, OR 97331, USA; 3 Department of Plant Production and Technologies, Faculty of Agricultural Sciences and Technology, Sivas Science and Technology University, Sivas 58000, Türkiye; kkokten@sivas.edu.tr; 4 Department of Agricultural Economics, College of Agriculture, Uludag University, Bursa 16059, Türkiye; abilgic@uludag.edu.tr

**Keywords:** Hungarian vetch, seed yield, GGE biplot analysis, genotype-environment interaction, yield components, climate resilience, sustainable agriculture

## Abstract

Climate change poses significant challenges to agriculture in Türkiye, where diverse climatic conditions demand resilient forage crops to meet rising roughage demands. This study evaluates the performance of Hungarian vetch (*Vicia pannonica* Crantz), a cold- and drought-tolerant legume, to enhance sustainable forage production. Eight genotypes (*Line-5*, *Line-16*, *Line-23*, *Line-28*, *Tarm White*, *Aegean White*, *Budak*, *and Oguz*) were tested in Bilecik and Bingol during the 2015–2016 and 2016–2017 growing seasons using a randomized complete block design with three replications. Key traits such as pods per plant, thousand-seed weight, biological yield, seed yield, straw yield, and harvest index were analyzed using Genotype plus Genotype-by-Environment (GGE) biplot analysis based on Principal Component Analysis (PCA). The distribution of these traits was also examined using box plots. Results showed significant variations: pods per plant ranged from 17.5 to 21.7, thousand-seed weight from 26.8 to 42.6 g, biological yield from 5710 to 8780 kg ha^−1^, seed yield from 826 to 1132 kg ha^−1^, straw yield from 4997 to 7643 kg ha^−1^, and harvest index (HI) from 13.9% to 21.0%. *Aegean White* excelled in seed yield, while *Line-16* showed the highest harvest index. GGE biplot analysis highlighted harvest index as the primary variance contributor, emphasizing genotype-environment interactions for selecting adaptable cultivars for sustainable agriculture.

## 1. Introduction

In the contemporary era, climate change has emerged as an urgent and formidable challenge, marked by the escalating consequences of global warming and cooling. These environmental shifts exert profound effects on agriculture and animal husbandry. In response to these pressing challenges, a key strategy for mitigation involves cultivating plant species and varieties that demonstrate resilience to these dynamic conditions. Despite the commendable support measures implemented in Türkiye since the early 2000s, which have significantly increased the sowing rate of forage crops [1], the production of high-quality roughage still falls short of meeting the growing demand. Unfortunately, only a limited number of forage crops, such as clover, sainfoin, vetch, and corn, have achieved widespread adoption in the country, while the considerable potential of many other forage crops remains unexplored. In this context, Hungarian vetch emerges as a promising candidate. This annual cool-season legume fodder plant exhibits remarkable resilience to both cold and drought, thriving even in challenging conditions such as heavy clay soils [2,3]. Its unique characteristics make it indispensable for expanding forage crop cultivation within Türkiye’s agricultural landscape. Notably, Hungarian vetch is the only annual legume fodder plant successfully integrated into the winter cropping systems practiced across diverse regions [4,5].

As of recent data, the cultivation area of Hungarian vetch (*Vicia pannonica* Crantz) in Türkiye is approximately 73,918 hectares, with about 7912 hectares dedicated to seed production [6]. Hungarian vetch is an important legume crop used primarily for forage and hay production, especially in the semi-arid regions of Central Anatolia, where it is often grown in rotation or mixed cropping with cereals such as oats and barley.

However, the country’s geographical regions are characterized by substantial climatic variations, which profoundly influence the performance of different plant species and varieties. Unfortunately, research efforts aimed at uncovering region-specific plant diversity remain relatively inadequate, primarily due to the complex climatic variations that span both inter- and intra-regional domains. Selecting plant species with broad adaptability is central to identifying the most suitable candidates for specific regional contexts, a challenge that demands focused attention. A key aspiration of plant breeders is to develop varieties capable of thriving across diverse environmental conditions, with a particular emphasis on vital traits such as yield [7]. Over time, plant breeders, geneticists, statisticians, and other field experts have integrated genetic (G) and genotype-by-environment (GE) interactions into their work (often abbreviated as GGE), aiming to select superior genotypes through crop performance trials [8,9]. A rich array of statistical methods has been developed for GGE interaction analysis, including the widely used Additive Main effects and Multiplicative Interaction (AMMI) analysis [8] and the influential GGE biplot analysis [10,11].

The escalating impacts of climate change, characterized by rising temperatures and increasingly erratic weather patterns, pose serious challenges to agriculture and livestock production worldwide [12]. In Türkiye, where agriculture plays a pivotal economic and social role [13,14], pressure is mounting on the country’s diverse agro-ecological zones, making the adoption of sustainable practices and the development of resilient crop varieties imperative. Despite government subsidy programs promoting the cultivation of forage crops, production of high-quality roughage remains insufficient, and use of alternative forage species is limited [15,16,17]. Hungarian vetch (*Vicia pannonica* Crantz), a cold- and drought-tolerant annual legume, has emerged as a promising candidate for diversifying forage resources, particularly in environmentally harsh regions [18,19]. As a vital component of winter cropping systems in Türkiye [20,21], its adaptability and yield potential must be evaluated in different climatic conditions due to significant regional variability. Recent advances in plant breeding methodologies, particularly GGE biplot analysis [14,15,16,17,18], which considers the interaction between genotype and environment, have been crucial for identifying stable, high-performing genotypes [8,10,11,15,17]. In response to these critical needs, the present study provides breeders, agronomists, and agricultural scientists with a practical guide that uses GGE biplot analysis to evaluate Hungarian vetch genotypes in two ecologically distinct regions: Bilecik and Bingol. The ultimate aim is to identify adaptable, high-yielding genotypes [22,23,24] that can support sustainable forage production in the face of intensifying climate change pressures [17].

## 2. Materials and Methods

This study was conducted simultaneously in two distinct ecological settings: the Bilecik region, representing the Eastern Marmara Region, and the Bingol region, representing the Eastern Anatolia Region, during the 2015–2016 and 2016–2017 growing seasons. The research material included Hungarian vetch lines (*Line-5*, *Line-16*, *Line-23*, and *Line-28*) as well as varieties developed by Bingol University’s College of Agriculture, Field Crops Department, including *Tarm White*, *Aegean White*, *Budak*, *and Oguz.*

The experimental plots were established in October, during winter, at the research and application areas of Bingol University’s Faculty of Agriculture and Bilecik Şeyh Edebali University. Hungarian vetch was sown manually on 10 October 2016, in Bilecik and on 12 October 2017, in Bingol during the first year of the study. In the second year, sowing took place on 15 October 2016, in Bilecik and on 17 October 2017, in Bingol. The seeding rate was 8 kg da^−1^ (≈80 kg ha^−1^), corresponding to approximately 280–300 seeds m^−2^, in accordance with regional recommendations for row planting under local ecological conditions. A randomized block design was used for the experiments, with three replications. Each experimental plot consisted of 5 rows, each 4 m long, with 0.30 m spacing between rows, resulting in a total plot area of 6 m^2^. Fertilization was applied at the time of sowing at a rate of 30 kg/ha of nitrogen (N) and 50–100 kg/ha of phosphorus pentoxide (P_2_O_5_), based on the soil test results and regional agronomic recommendations.

**Figure 1:**
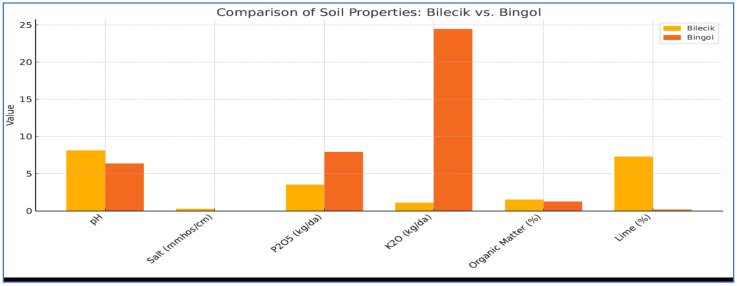
Some physical and chemical properties of the soils of the trial areas.

The soil properties of the research sites are summarized in Figure 1, with technical term abbreviations provided upon their first mention.

The soil in the trial area of Bilecik is predominantly sandy and loamy, with a moderately alkaline pH of 8.11 and moderate salinity at 0.26 mmhos/cm. It contains moderate levels of lime (7.3%) and organic matter (1.5%), along with lower concentrations of phosphorus (3.5 kg da^−1^ P_2_O_5_, low level) and potassium (1.1 kg da^−1^ K_2_O, very low level), though sufficient quantities of essential microelements are present. In contrast, the soil in the experimental area of Bingol is primarily loamy, with a slightly acidic pH of 6.37 and minimal salt content of 0.0006 mmhos/cm. Its organic matter content is relatively low at 1.26%, while phosphorus levels are moderate (7.9 kg da^−1^ P_2_O_5_, medium level) and potassium content is very high (24.45 kg da^−1^ K_2_O, very high level). Additionally, the Bingol soil exhibits lower calcareous properties.

According to the data obtained from the Bilecik Provincial Meteorology Station, during the 2016–2017 research period, the average temperatures for the months of September, October, November, December, January, February, March, April, May, and June were recorded as 25.6 °C, 20.0 °C, 13.6 °C, 7.8 °C, 6.2 °C, 8.2 °C, 12.1 °C, 17.3 °C, 22.6 °C, and 22.5 °C, respectively. The total precipitation for these months was 26.7 mm, 51.6 mm, 39.0 mm, 54.0 mm, 46.6 mm, 43.8 mm, 50.2 mm, 49.6 mm, 41.2 mm, and 69.9 mm, respectively, while the relative humidity values were 64.8%, 70.5%, 71.9%, 76.2%, 76.4%, 73.0%, 68.3%, 64.9%, 63.2%, and 67.3%, respectively. Similarly, data from the Bingol Meteorology Station for the same 2016–2017 research period revealed the following average temperatures for the months from September to June: 23.4 °C (September), 14.3 °C (October), 14.4 °C (November), 1.3 °C (December), −1.8 °C (January), 1.9 °C (February), 5.4 °C (March), 10.9 °C (April), 16.6 °C (May), and 22.9 °C (June). The total precipitation for these months was recorded as 0.8 mm, 220.9 mm, 18.9 mm, 46.2 mm, 148.2 mm, 115.8 mm, 154.4 mm, 66.7 mm, 21.2 mm, and 8.1 mm, respectively. Relative humidity values were 30.2%, 68.3%, 56.4%, 58.6%, 74.7%, 73.8%, 65.9%, 58.7%, 52.0%, and 37.0%, respectively.

Morphological and phenological observations of the plants were conducted when the lower fruits reached maturity. For seed yield and yield component analysis, harvesting was performed once the pods had completely turned yellow. Technical terms were introduced and explained as needed. The average number of pods per plant (number/plant) was calculated by counting the pods on 10 randomly selected plants from each plot. Harvested parcels were divided into seed yield and biological yield (kg da^−1^) for further analysis. Hungarian vetch seeds were harvested at full maturity on 5 July 2021, in Bilecik and on 12 July 2021, in Bingol during the first year, and on 8 July 2022, in Bilecik and on 15 July 2022, in Bingol during the second year of the study. Biological yield was determined by measuring the dry weight of the total above-ground plant biomass (including seeds, stems, leaves, and other plant parts) harvested from each plot. The plants were first weighed fresh at harvest, then dried in a laboratory oven at 70 °C for 48 h to obtain constant dry weight. These values were obtained directly from field measurements and do not represent theoretical calculations. Seeds were then extracted, counted, and the number of seeds per pod was determined by dividing the total number of seeds by the total number of pods. In the present study, the first two principal components accounted for 79% of the total variation, which is considered sufficiently high to capture the main patterns in genotype × environment interactions. Following standard practice in PCA and GGE biplot analysis, when the cumulative variance explained by the first two components exceeds approximately 70%, these components are generally regarded as adequate for reliable interpretation.

## 3. Results and Discussion

The mean differences between the eight genotypes were determined for each measured trait in Hungarian vetch, with analysis conducted separately for each year and location. This was achieved using the least significant difference (LSD) method. Alphabetical lettering was then used to indicate statistical groupings. Next, analysis of variance (ANOVA) was performed within each location to evaluate the statistical significance of the interactions between genotype and year. A combined ANOVA was then conducted to assess the significance of the three-way interaction between genotype, location, and year. Additionally, the statistical significance of genotype × location interactions was examined separately for each year and across both years using ANOVA. The statistical data calculated for traits such as the number of pods per plant, thousand-seed weight (g), biological yield (kg da^−1^), seed yield (kg da^−1^), straw yield (kg da^−1^), and harvest index (%) are presented in Table 1, Table 2, Table 3, Table 4, Table 5 and Table 6, respectively. Detailed explanations for each table are provided below in the same order.

Table 1 outlines the variation in pod number per plant among Hungarian vetch (*Vicia pannonica*) genotypes across two distinct environments, Bilecik and Bingol, over the 2015–2016 and 2016–2017 growing seasons. The data reveal clear and meaningful differences in performance linked to genotype, year, and location, emphasizing both the environmental responsiveness of this species and the role of genetic diversity in yield expression. The number of pods per plant is a morphological trait that directly affects seed yield [22]. Among the genotypes tested, *Line-5* consistently stood out with the highest average pod number (21.7), showcasing superior and stable productivity. On the opposite end, the *Tarım White* genotype demonstrated the lowest yield (17.5 pods on average), highlighting how genetic potential is shaped by environmental interactions. Local environmental effects were particularly pronounced: for instance, in Bilecik, the *Oguz* genotype produced the highest yields in both years (26.7 and 22.9 pods), while *Budak* recorded the lowest. Meanwhile, in Bingol, *Line-28* excelled in 2015–2016 and *Line-5* in 2016–2017, whereas *Oguz* and *Tarım White* underperformed. Taken together, these findings position *Line-5* as a robust, high-yielding genotype with wide adaptation potential, while suggesting that *Oguz* may be less suited to the conditions in Bingol. The findings of this study align with previous literature. For example, [25] reported that the number of pods per plant ranged between 6.03 and 9.37 in a study conducted to determine the adaptation of some Hungarian vetch lines and varieties to the dry conditions of Bingol. Similarly, [26] found that the number of pods per plant ranged between 18.0 and 25.8 in a study evaluating Hungarian vetch varieties as bee pastures. Furthermore, other studies reported the number of pods per plant for different Hungarian vetch genotypes to range from 17.20 to 24.35 [27], 7.5 [28], and 8.93 to 24.93 [29]. Further studies focusing on its physiological and biochemical traits could offer deeper insight into its stress tolerance and long-term agronomic value.

**Table 1:** Pod numbers of per plant (number) of Hungarian vetch at both locations.

Genotypes	Locations						Year		Overall Mean
	Bilecik			Bingol			2015– 2016	2016– 2017	
	2015– 2016	2016– 2017	Mean Loc.	2015– 2016	2016– 2017	Mean Loc.			
*Tarm White*	25.7 a	18.1 b	21.9 a	12.3 cd	14.0 d	13.2 d	19.0 a	16.1 c	17.5 b
*Budak*	23.4 a	20.1 ab	21.7 a	14.3 abcd	17.3 bc	15.8 bcd	18.9 a	18.7 abc	18.8 ab
*Line-16*	26.1 a	21.2 ab	23.7 a	16.3 abcd	17.7 bc	17.0 abc	21.2 a	19.4 ab	20.3 ab
*Aegean White*	24.1 a	20.6 ab	22.3 a	13.3 bcd	15.0 cd	14.2 cd	18.7 a	17.8 bc	18.2 ab
*Oguz*	26.7 a	22.9 a	24.8 a	11.7 d	14.3 d	13.0 d	19.2 a	18.6 abc	18.9 ab
*Line-23*	26.4 a	22.9 a	24.6 a	18.0 ab	19.0 ab	18.5 ab	22.2 a	20.9 ab	21.6 a
*Line-28*	25.0 a	20.1 ab	22.5 a	18.3 a	20.7 a	19.5 a	21.7 a	20.4 ab	21.0 ab
*Line-5*	25.8 a	22.2 a	24.0 a	17.0 abc	21.7 a	19.3 a	21.4 a	21.9 a	21.7 a
**Overall mean**	25.4	21.0	23.2	15.2	17.5	16.3	20.3	19.2	19.8
Aov (Gen.)	0.91 (*p* = 0.52)	2.35 * (*p* = 0.08)	0.96 (*p* = 0.47)	2.65 ** (*p* = 0.05)	9.66 *** (*p* = 0.00)	7.15 *** (*p* = 0.00)			
Aov (Gen.xYear)	0.78 (*p* = 0.61)			0.41 (*p* = 0.89)					
**Statistical information about three way interaction:**									
Aov (Gen.xLoc.)	4.77 *** (*p* = 0.00)						1.88 (*p* = 0.11)	3.99 *** (*p* = 0.00)	2.79 ** (*p* = 0.01)
Aov (Gen.xYear)	0.74 (*p* = 0.64)								
Aov (Loc.xYear)	59.03 *** (*p* = 0.00)								
Aov (Gen.xLoc.xYear)	0.40 (*p* = 0.899)								

Note: *, **, and *** show statistical significance levels at 10%, 5%, and 1%, respectively. Also, means followed by different letters in the same column are significantly different according to the Least Significant Difference (LSD) test at *p* < 0.05 while the same letter indicates no statistically significant difference. Mean Loc. shows the mean locations while Aov stands for analysis of variance.

These observations are statistically reinforced by the results of the variance analysis. Genotypic differences were significant in Bingol (*p* < 0.05 and *p* < 0.001 across the two years), indicating a strong environmental influence on performance in this location. In contrast, genotype-related differences in Bilecik were not significant (*p* > 0.05), implying a more uniform response across genotypes. Particularly noteworthy is the highly significant genotype × location interaction (*p* < 0.001), which underscores that genotypic performance is heavily context-dependent and that success in one location does not necessarily translate to broader adaptability. While genotype × year and location × year interactions were non-significant (*p* > 0.05), suggesting a degree of annual stability, the significant three-way interaction (genotype × location × year, *p* < 0.01) points to a more complex interplay of genetic and environmental factors. This emphasizes the need to consider both stability and responsiveness in breeding programs aimed at improving adaptability under varying conditions.

**Figure 2:**
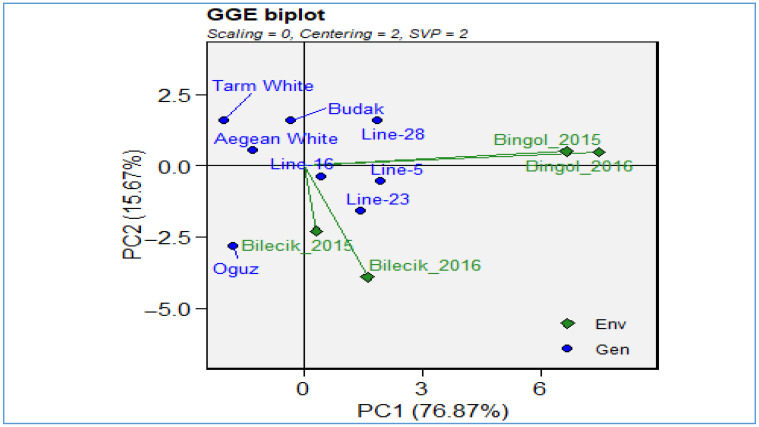
GGE biplot for pods per plant.

To more effectively interpret these complex interactions, a GGE biplot analysis (Figure 2) was employed, offering a visual assessment of both genotypic main effects and their interactions with the environment. The first two principal components explained 92.54% of the total variation, validating the robustness of the biplot. While the first two principal components accounted for 79% of the total variation, thereby providing a robust basis for genotype × environment interaction analysis, it is important to acknowledge that the exclusion of higher-order components may result in overlooking patterns of smaller magnitude but potential biological relevance. These components, although explaining a relatively minor proportion of variance individually, could reflect specific environmental responses or trait associations not captured in the first two dimensions. Therefore, this should be considered a limitation when interpreting the results of the present study.

In the plot, environments are shown as green diamonds and genotypes as blue circles. The close clustering of Bingol years suggests stable environmental conditions and consistent genotypic responses, whereas the wider dispersion of Bilecik years reflects environmental variability. Genotypes such as *Line-5*, *Line-23*, and *Line-28* were centrally positioned, indicating both high performance and stability across environments. In particular, *Line-5* demonstrated a strong balance between yield and adaptability, reinforcing its value as a strategic candidate in breeding programs. Conversely, genotypes like *Tarım White*, *Aegean White*, and *Budak* were located at more distant points on the biplot, revealing variable and environment specific responses. Ultimately, the GGE biplot proved to be a powerful tool for evaluating environmental selectivity and genotypic stability in multi-environment trials. *Line-5*, by combining high yield potential with environmental resilience, stands out as a key candidate for future cultivar development in the context of climate change and sustainability.

**Figure 3:**
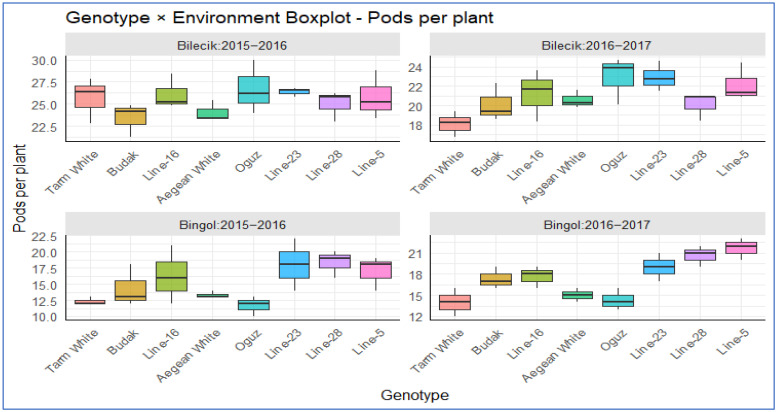
The distribution of pod numbers per plant across different genotypes in two distinct locations.

Figure 3 presents a biplot analyzing the distribution of pod counts across different genotypes. In Bilecik, *Oguz* and *Tarm White* notably excelled in the 2015–2016 season, achieving pod counts of approximately 25–27 per plant, surpassing their peers. In contrast, *Line-23* exhibited the lowest pod count distribution, maintaining stable performance throughout. Fast forward to the 2016–2017 season, and the *Oguz* and *Line-23* genotypes surged ahead, achieving consistent yields of 22–24 pods per plant. In contrast, the *Tarm White* and *Budak* genotypes underperformed relative to the group, achieving yields of 18–20 pods per plant. *Aegean White* displayed the lowest pod count distribution this season, highlighting its difficulty in adapting to the conditions in Bilecik. In Bingol, *Line-23* and *Line-28* took the lead in the 2015–16 season with 17–19 pods per plant, while *Tarm White* and *Aegean White* trailed behind with more modest outputs of 12–15 pods per plant. Interestingly, *Line-23* and *Line-16* exhibited the greatest variability, while *Tarm White* maintained a consistent distribution. By the 2016–2017 season, the distribution in Bingol had become more balanced. *Line-5*, *Line-28*, and *Line-23* led the pack with 18–22 pods per plant, with an overall average of 15–18 pods. *Aegean White* recorded the lowest yield again, though other genotypes demonstrated a remarkably homogeneous distribution. A cross-location comparison reveals that Bilecik is a more favorable environment, supporting pod counts of 20–26, whereas Bingol’s potential is more limited, with an average of 12–21 pods per plant. This suggests that environmental factors play a pivotal role in shaping genotype performance.

As demonstrated in Table 2, the thousand-grain weight measurements (in grams) for various Hungarian vetch genotypes were documented across two growing seasons (2015–2016 and 2016–2017) at the Bilecik and Bingol locations. The thousand-grain weight is a critical morphological trait that exerts a direct influence on seed size, yield potential, and overall agronomic performance. This factor is well-established in the literature as a key determinant of germination rates, seedling vigor, and seed yield, making it an essential indicator of crop productivity and quality [27]. The data reveal highly significant variations (*p* < 0.01) in thousand-grain weights among genotypes, years, and locations, along with notable genotype × year, genotype × location, and location × year interactions. These findings emphasize the considerable impact of genetic diversity and environmental variability on trait expression, providing significant insights into the genotype-by-environment (G × E) interactions that influence agricultural performance. Statistical analysis confirms highly significant genotypic effects (*p* < 0.01) and pronounced location-specific responses, with ANOVA results validating these observations. The significant interactions demonstrate the complexity of G × E effects, indicating that the performance of Hungarian vetch is contingent on both genetic potential and environmental conditions. Such interactions are of critical importance in the development of breeding programs that are aimed at cultivating varieties that exhibit enhanced adaptability and stability across diverse agro-ecological zones. The dataset, which has been collected across multiple seasons and locations, provides a robust foundation for these analyses.

**Table 2:** Thousand-seed weight (g) of Hungarian vetch at both locations.

Genotypes	Locations						Year		Overall Mean
	Bilecik			Bingol			2015– 2016	2016– 2017	
	2015– 2016	2016– 2017	Mean Loc.	2015– 2016	2016– 2017	Mean Loc.			
*Tarm White*	38.5 b	35.5 ab	36.9 bc	41.3 a	34.6 b	37.9 b	39.9 a	35.0 b	37.4 b
*Budak*	35.7 cd	33.5 c	34.6 de	34.5 b	34.1 b	34.3 c	35.1 b	33.8 b	34.4 c
*Line-16*	33.2 e	31.4 d	32.3 f	33.6 b	27.0 d	30.3 d	33.4 bc	29.2 c	31.3 de
*Aegean White*	36.4 c	34.5 bc	35.4 cd	34.0 b	33.1 b	33.6 c	35.2 b	33.8 b	34.5 c
*Oguz*	39.4 b	37.1 a	38.2 ab	42.1 a	43.2 a	42.6 a	40.7 a	40.1 a	40.4 a
*Line-23*	34.0 e	31.6 d	32.8 ef	30.6 c	25.8 d	28.2 de	32.3 bc	28.7 c	30.5 de
*Line-28*	41.5 a	36.9 a	39.2 a	27.4 d	26.4 d	26.8 e	34.4 b	31.6 bc	33.0 cd
*Line-5*	34.3 de	29.5 e	31.9 f	25.3 d	29.3 c	27.3 e	29.8 c	29.4 c	29.6 e
Overall mean	36.6	33.7	35.2	33.6	31.7	32.6	35.1	32.7	33.9
Aov (Gen.)	33.0 *** (*p* = 0.00)	20.3 *** (*p* = 0.00)	12.8 *** (*p* = 0.00)	72.0 *** (*p* = 0.00)	67.87 *** (*p* = 0.00)	30.6 *** (*p* = 0.00)			
Aov (Gen.xYear)	2.19 * (*p* = 0.06)			14.56 *** (*p* = 0.00)					
**Statistical information about three way interaction:**									
Aov (Gen.xLoc.)	60.27 *** (*p* = 0.00)						46.04 *** (*p* = 0.00)	28.05 *** (*p* = 0.00)	15.13 *** (*p* = 0.00)
Aov (Gen.xYear)	7.15 *** (*p* = 0.00)								
Aov (Loc.xYear)	4.35 ** (*p* = 0.04)								
Aov (Gen.xLoc.xYear)	12.43 *** (*p* = 0.00)								

Note: *, **, and *** show statistical significance levels at 10%, 5%, and 1%, respectively. Also, means followed by different letters in the same column are significantly different according to the Least Significant Difference (LSD) test at *p* < 0.05 while the same letter indicates no statistically significant difference. Mean Loc. shows the mean locations while Aov stands for analysis of variance.

**Figure 4:**
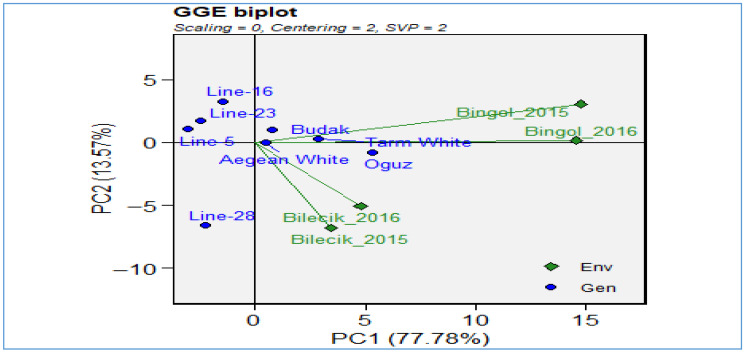
GGE biplot for thousand seed weight.

As shown in Figure 4, a GGE biplot provides a comprehensive visual assessment of genotypic main effects and genotype-environment interactions, thereby facilitating interpretation of complex multi-environment dynamics. The initial two principal components (PC1 and PC2) account for 91.35% of the total variation, thereby demonstrating the efficacy of the GGE biplot in depicting salient data patterns. The tight clustering of Bingol environments observed across both years indicates consistent environmental conditions and stable genotypic responses. Conversely, the broader dispersion of Bilecik environments reflects greater environmental variability and more diverse conditions influencing genotype performance. Genotypes positioned centrally—*Line-5*, *Line-23*, and *Line-28*—have been demonstrated to exhibit both superior performance and stability across a range of tested environments. *Line-5* demonstrates particular excellence in its optimal balance of yield potential and adaptability, thus establishing it as a valuable candidate for breeding programs focused on resilience and productivity. In contrast, genotypes located peripherally, such as *Tarm White*, *Aegean White*, and *Budak*, exhibit environment-specific responses that have the potential to compromise their stability across diverse conditions. The GGE biplot is a useful tool for the effective evaluation of environmental selectivity and genotypic stability in multi-environment trials. The findings are supported by analysis of two-year location data. The *Oguz* genotype demonstrated the highest thousand-grain weight at Bingol (42.6 g), while *Line-28*, *Line-5*, and *Line-23* exhibited the lowest values at the same location (26.8 g, 27.3 g, and 28.2 g, respectively). These results underscore the environmental influences on genotypic expression. The present study has demonstrated that comparative literature serves to reinforce the observed variability in thousand-seed weight among Hungarian vetch accessions. Research conducted in Bingol’s arid environment has documented ranges of 32.60–40.90 g [28], while evaluations of bee pastures have shown 26.3–38.6 g [29]. Further research documented ranges of 32.08–39.15 g [30] and broader variations from 17.3 to 34.7 g [31]. Furthermore, comprehensive assessments of seed and straw yield revealed that thousand-seed weights ranged from 25.32 to 43.23 g [32]. These findings corroborate the GGE biplot results, emphasizing genetic diversity and environmental factors as key determinants of thousand-seed weight and positioning *Line-5* as a promising candidate for future cultivar development in the context of climate adaptation and sustainable agriculture.

**Figure 5:**
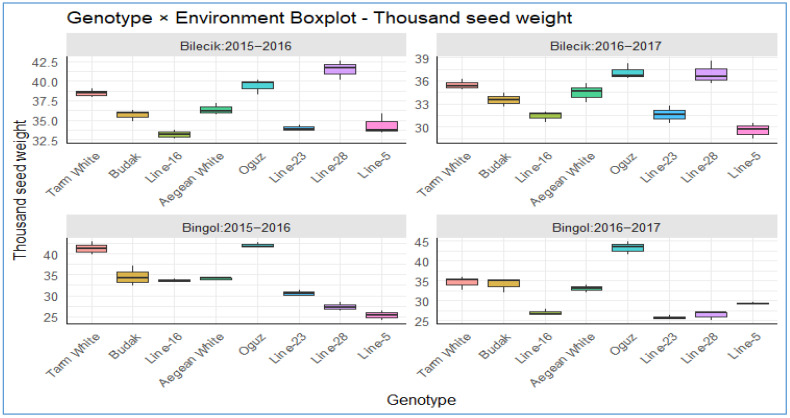
The distribution of thousand seed weight across different genotypes in two distinct locations.

Figure 5 shows striking genotype-environment interactions in Hungarian vetch thousand-seed weights, with performance patterns exhibiting both compelling stability and concerning volatility across locations and years. While *Tarm White*, *Budak*, and *Aegean White* have been demonstrated to demonstrate exceptional phenotypic buffering with consistent 32–45 g performance across all four environments, thus indicating robust genetic canalization mechanisms, genotypes such as *Oguz* and *Line-28* have been shown to display dramatic environmental sensitivity, ranging from exceptional performance (>42 g) to concerning lows (<27 g). A particularly noteworthy finding is the environmental stress signature observed in Bingol 2016–2017, where multiple genotypes (*Line-28*, *Line-5*, and *Line-23*) exhibited significant performance reductions to 26–28 g, while *Oguz* paradoxically demonstrated peak performance. This finding indicates the existence of genotype-specific stress tolerance mechanisms that contradict conventional stability assumptions. The contrasting performance ceilings observed between the Bilecik and Bingol environments, which are generally higher in the former, coupled with the pronounced year effects, underscore the complexity of genotype × environment × year interactions. These interactions have the potential to complicate the strategy of breeding programs. This is primarily due to the fact that agricultural systems are progressively requiring both high performance and predictable stability in the face of climate variability. Consequently, genotypes demonstrating moderate performance but low variability are potentially more valuable than high-performing but unstable alternatives.

As presented in Table 3, the total above-ground biological yield (kg ha^−1^) is a pivotal indicator of productivity and adaptability for Hungarian vetch genotypes. A thorough statistical analysis has been conducted, unveiling profoundly significant disparities (*p* < 0.01) in biological yield across a range of parameters, encompassing years, locations, and location-year interactions. This analysis underscores the substantial impact of genotype-by-environment (G × E) interactions, emphasizing the necessity for a comprehensive understanding of biological processes under diverse environmental conditions. Despite the yearly means being found to be insignificant (*p* > 0.05), indicating temporal stability, the *Aegean White* genotype achieved the highest yield (1039.9 kg ha^−1^) at Bingol in 2015–2016, whereas *Line-23* recorded the lowest (552.0 kg ha^−1^) at the same site. A significant genotypic variation was observed in both locations (*p* < 0.01). A robust genotype × location interaction (*p* < 0.001) and a significant three-way interaction (*p* < 0.01) were identified, suggesting that performance is context-dependent. The two-year location mean data further demonstrates that *Aegean White* is leading at 774.3 kg ha^−1^, while *Line-16* is trailing at 594.3 kg ha^−1^. This emphasizes the variability in biological yield and highlights the necessity for breeding programs that prioritize stability. A substantial body of comparative research has documented biological yields ranging from 1880 to 2640 kg ha^−1^ [33], thereby emphasizing the species’ environmental sensitivity and adaptability.

**Table 3:** Biological yield (kg ha^−1^) of Hungarian vetch at both locations.

Genotypes	Locations						Year		Overall Mean
	Bilecik			Bingol			2015– 2016	2016– 2017	
	2015– 2016	2016– 2017	Mean Loc.	2015–2016	2016–2017	Mean Loc.			
*Tarm White*	760.7 a	627.1 b	693.9 a	829.3 d	762.6 b	795.9 ab	794.9 a	6948 ab	744.9 ab
*Budak*	717.4 ab	693.6 a	705.5 a	883.2 c	648.7 de	765.9 ab	800.3 a	6711 ab	735.7 ab
*Line-16*	567.1 d	587.3 c	577.2 b	640.7 e	582.0 fg	611.4 cd	603.9 c	5847 c	594.3 c
*Aegean White*	677.4 bc	663.8 a	670.6 a	1039.9 a	716.1 c	878.0 a	858.7 a	6899 ab	774.3 a
*Oguz*	624.0 cd	604.0 bc	614.0 b	645.2 e	840.9 a	743.0 abc	634.7 bc	7225 a	678.5 bc
*Line-23*	664.1 bc	690.3 a	677.2 a	817.5 d	552.0 g	684.8 bcd	740.8 ab	621.2 bc	681.0 bc
*Line-28*	700.7 abc	667.1 a	683.9 a	984.9 b	683.7 cd	834.3 a	842.8 a	675.4 ab	759.1 ab
*Line-5*	710.5 ab	689.8 a	700.2 a	519.0 f	623.1 ef	571.0 d	614.8 bc	656.5 abc	635.6 c
**Overall mean**	677.8	652.9	665.3	795.0	676.1	735.5	736.4	664.5	700.5
Aov (Gen.)	48.0 *** (*p* = 0.00)	163.0 *** (*p* = 0.00)	67 *** (*p* = 0.00)	1297 *** (*p* = 0.00)	463.0 *** (*p* = 0.00)	4.6 *** (*p* = 0.00)			
Aov (Gen.xYear)	27.5 ** (*p* = 0.02)			84.37 *** (*p* = 0.00)					
**Statistical information about three way interaction:**									
Aov (Gen.xLoc.)	324.3 *** (*p* = 0.00)						282.5 *** (*p* = 0.00)	464.6 *** (*p* = 0.00)	38.7 *** (*p* = 0.00)
Aov (Gen.xYear)	286.4 *** (*p* = 0.00)								
Aov (Loc.xYear)	536.4 *** (*p* = 0.00)								
Aov (Gen.xLoc.xYear)	324.7 *** (*p* = 0.00)								

Note: **, and *** show statistical significance levels at 5%, and 1%, respectively. Also, means followed by different letters in the same column are significantly different according to the Least Significant Difference (LSD) test at *p* < 0.05 while the same letter indicates no statistically significant difference. Mean Loc. shows the mean locations while Aov stands for analysis of variance.

**Figure 6:**
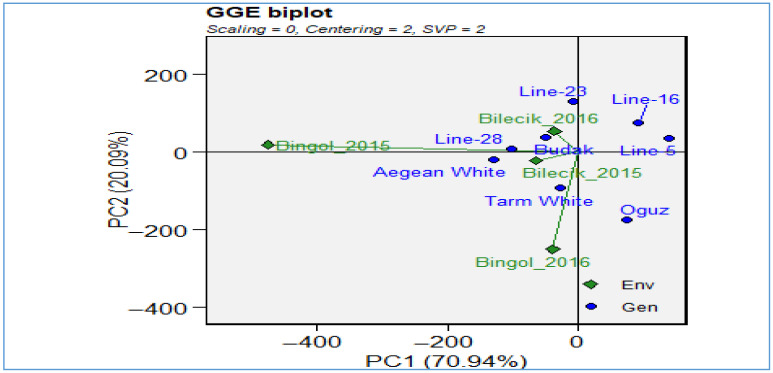
GGE biplot for biological yield.

As demonstrated in Figure 6, the interaction between genotypes (blue points) and environments (green diamonds) in terms of biological yield performance is illustrated using a GGE biplot. The horizontal axis (PC1) accounts for 70.94% of the total variance, while the vertical axis (PC2) is responsible for 20.09%, thus collectively explaining over 91% of the total variation—an exceptionally high degree of explanatory power. The environments encompass Bilecik 2015 and 2016 and Bingol 2015 and 2016, which collectively represent the influence of disparate years and locations on yield performance. The genotypes, represented by the blue color scheme, are distributed in relation to these environments, thereby revealing which cultivars perform optimally under specific conditions. Genotypes positioned further to the right along PC1 generally exhibit higher mean yields, whereas their position on PC2 reflects yield stability; those closer to the horizontal axis tend to be more stable across environments. For instance, the upper-right quadrant’s *Line-16* and *Line-5* exhibit a combination of elevated yield potential and the potential for adaptation benefits in particular conditions. In contrast, genotypes positioned towards the center of the plot, including *Aegean White*, *Line-28*, *Budak*, and *Tarm White*, exhibited enhanced stability but demonstrated comparatively diminished average yields. The proximity of environmental locations is indicative of similar climatic or management conditions; for instance, the close proximity of Bilecik 2015 and Bilecik 2016 suggests similar influences on genotype performance. Conversely, Bingol 2015 is positioned to the left, suggesting unique circumstances and interactions between genotype and environment. When all factors are taken into consideration, the biplot provides an effective visual aid that facilitates the identification of genotypes that demonstrate high performance in specific environments. Furthermore, it facilitates the identification of commonalities between test environments and the investigation.

**Figure 7:**
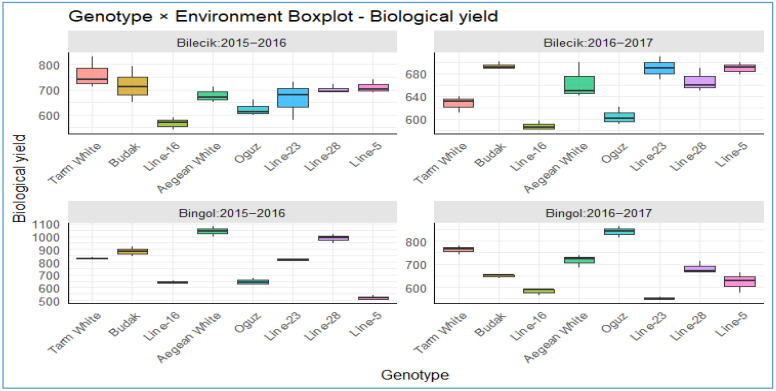
The distribution of biological yield across different genotypes in two distinct locations.

The boxplots in Figure 7 present a striking visual narrative of the performance of diverse genotypes under the sharply contrasting climatic realities of Bilecik and Bingol during the 2015–2016 and 2016–2017 seasons. This narrative underscores the pivotal role of genotype–environment interactions in shaping agricultural sustainability. In Bilecik’s temperate, humid environment, except the consistently low-yielding *Line-16* and the notably high-performing *Tarm White*, the majority of genotypes exhibited remarkably consistent and substantial yields within the range of 700–800 kg da^−1^. Of particular interest are *Budak*, *Aegean White*, *Line-28*, and *Line-25*, which exhibit narrow box widths and low variability, indicative of their remarkable resilience to climatic variations. The performance of these lines is characterized by consistency, with a stable yield maintained at a rate of approximately 650–700 kg da^−1^. Conversely, Bingol’s more arid conditions resulted in a substantial decline in yield, typically to 600–700 kg da^−1^. However, this environment also revealed notable exceptions, with genotypes such as *Aegean White* and *Oguz* achieving remarkable yields of 900–1050 kg da^−1^. These exceptions demonstrated a notable sensitivity to environmental stress. The persistent underperformance of *Line-16* in both locations suggests the presence of genetic constraints, while the observed season-to-season improvement in Bilecik by *Line-5* indicates the potential for adaptive responses. However, this trend is less apparent in Bingol. When considered collectively, these results provide a compelling argument from the perspective of modern agronomy that in the era of climate change, the strategic selection of resilient genotypes is not merely advantageous but essential for safeguarding yield potential and reinforcing agricultural resilience.

Table 4 elicits the seed yield (kg ha^−1^) of Hungarian vetch genotypes across the 2015–2016 and 2016–2017 seasons at Bilecik and Bingol, serving as a key metric of reproductive success and environmental adaptability. Statistical analysis reveals significant genotypic differences (*p* < 0.01) and a highly significant genotype × location interaction (*p* < 0.001), underscoring the influence of genetic diversity and environmental conditions on yield expression. However, mean values across years were not statistically significant (*p* > 0.05), indicating temporal stability. The *Aegean White* genotype excelled with the highest location-year interaction yield of 126.7 kg ha^−1^ at Bingol in 2016–2017, followed by *Line-16* and *Line-28* at Bilecik in 2015–2016 with 124.0 and 123.3 kg ha^−1^, respectively. Conversely, the lowest yield was recorded for *Line-23* at Bingol in 2015–2016, at 37.5 kg ha^−1^. Over the two-year location mean, *Aegean White* achieved the highest seed yield at 113.2 kg ha^−1^, while *Line-23* recorded the lowest at 82.6 kg ha^−1^. This variability highlights genotype-specific responses to diverse edaphic, biotic, and abiotic factors, consistent with findings that environmental heterogeneity drives differential performance [34,35].

Climatic conditions varied markedly between the two research sites, with Bingol experiencing more extreme winter temperatures and less favorable humidity patterns during critical growth stages. In Bingol, average temperatures dropped to 1.3 °C in December and −1.8 °C in January, with relative humidity falling to 30.2% in September and 37.0% in June. Such cold winters can lead to partial winterkill and slower regrowth in spring, while low humidity during establishment and maturation phases may exacerbate plant water stress. Additionally, Bingol’s rainfall pattern was irregular, with very low precipitation in September (0.8 mm) followed by excessive rainfall in October (220.9 mm), which could have caused both early drought stress and subsequent waterlogging. In contrast, Bilecik exhibited milder winter temperatures (7.8 °C in December and 6.2 °C in January), higher relative humidity (64.8–76.4% across the winter months), and more balanced rainfall distribution, which likely promoted better seedling establishment, sustained vegetative growth, and higher biomass yield. These distinct climatic regimes help explain the significant location × genotype interactions observed in this study. These results position *Aegean White* as a promising candidate for breeding programs targeting high and stable yields across varied conditions.

**Table 4:** Seed yield (kg ha^−1^) of Hungarian vetch at both locations.

Genotypes	Locations						Year		Overall Mean
	Bilecik			Bingol			2015– 2016	2016– 2017	
	2015– 2016	2016– 2017	Mean Loc.	2015– 2016	2016– 2017	Mean Loc.			
*Tarm White*	122.0 ab	90.1 c	106.0 a	55.9 d	85.3 cd	70.6 bc	88.9 a	87.7 d	88.3 bc
*Budak*	122.0 ab	98.0 abc	110.0 a	61.0 cd	102.0 b	81.5 bc	91.5 a	100.0 bc	95.8 abc
*Line-16*	124.0 a	101.6 abc	112.8 a	87.1 b	93.7 bc	90.4 ab	105.5 a	97.6 bcd	101.6 ab
*Aegean White*	122.3 ab	103.2 ab	112.8 a	100.7 a	126.7 a	113.7 a	111.5 a	114.9 a	113.2 a
*Oguz*	102.3 b	96.4 abc	99.4 a	57.6 d	117.0 a	87.3 b	79.9 a	106.7 ab	93.3 bc
*Line-23*	118.7 ab	92.4 bc	105.6 a	37.5 e	82.0 d	59.7 c	78.1 a	87.2 d	82.6 c
*Line-28*	123.3 a	105.5 a	114.4 a	65.8 c	88.7 cd	77.3 bc	94.6 a	97.1 bcd	95.8 abc
*Line-5*	119.7 ab	98.1 abc	108.9 a	55.8 d	87.0 cd	71.4 bc	87.7 a	92.5 cd	90.1 bc
**Overall mean**	119.3	98.2	108.7	65.2	97.8	81.5	92.2	97.9	95.1
Aov (Gen.)	1.0 (*p* = 0.44)	1.8 (*p* = 0.15)	0.7 (*p* = 0.71)	122.5 *** (*p* = 0.00)	20.1 *** (*p* = 0.00)	3.9 *** (*p* = 0.00)			
Aov (Gen.xYear)	0.9 (*p* = 0.52)			154.1 *** (*p* = 0.00)					
**Statistical information about three way interaction:**									
Aov (Gen.xLoc.)	12.00 *** (*p* = 0.00)						6.89 *** (*p* = 0.00)	8.1 *** (*p* = 0.00)	2.27 ** (*p* = 0.04)
Aov (Gen.xYear)	5.13 *** (*p* = 0.00)								
Aov (Loc.xYear)	291.62 *** (*p* = 0.00)								
Aov (Gen.xLoc.xYear)	2.65 ** (*p* = 0.02)								

Note: **, and *** show statistical significance levels at 5%, and 1%, respectively. Also, means followed by different letters in the same column are significantly different according to the Least Significant Difference (LSD) test at *p* < 0.05 while the same letter indicates no statistically significant difference. Mean Loc. shows the mean locations while Aov stands for analysis of variance.

The research highlights the significant impact of genotype-environment (G × E) interactions on seed yield in Hungarian vetch, a finding corroborated by studies on various forage crops [35,36,37]. G × E interactions, particularly genotype-location and genotype-location-year effects, proved more influential than genotype-year interactions [38,39], aligning with our results. Consistent with these observations, [40] reported seed yields of 12 Hungarian vetch genotypes ranging from 0.46 to 1.10 t ha^−1^ across five locations in Southeastern Anatolia, Türkiye, while [37,41] noted yields of seven genotypes between 0.54 and 0.76 t ha^−1^ in the Thrace Region.

**Figure 8:**
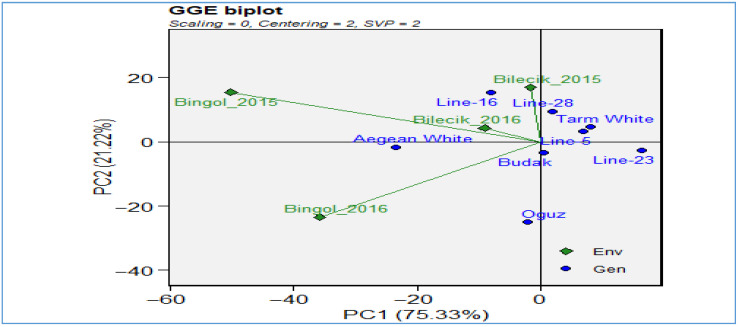
GGE biplot for seed yield.

Figure 8 GGE biplot for seed yield (kg da^−1^) provides an integrated view of genotype (blue points) and environment (green diamonds) interactions, revealing how performance varies across Bilecik 2015, Bilecik 2016, Bingol 2015, and Bingol 2016. The first principal component (PC1) accounts for the majority of the total variation, explaining 75.33% of the observed differences in yield among the genotypes. The second principal component (PC2) accounts for 21.22% of the variation, and it is associated with stability and interaction effects. Genotypes positioned further to the right along PC1 generally exhibited higher mean seed yields, while proximity to the plot origin suggested average performance with potential stability across environments. For instance, *Line-28*, *Line-16*, and *Tarm White*, which are located in close proximity to Bilecik 2015 and 2016, appear to be well adapted to the growing conditions in Bilecik, potentially benefiting from the region’s more temperate and humid climate. In contrast, the Bingol 2015 and 2016 environments are positioned further to the left, indicating disparate growing conditions—likely more arid or inhospitable—resulting in distinct genotype responses. Genotypes such as *Oguz* and *Line-23*, which are distant from the Bingol vectors, may exhibit reduced compatibility with the environmental conditions of Bingol. In contrast, the intermediate positions of *Aegean White* and *Budak* indicate a certain degree of adaptability. The distance and angle between environmental vectors indicate that Bilecik environments are more similar to each other, whereas Bingol 2015 and 2016 differ not only from Bilecik but also from each other, hinting at inter-annual climatic variation. The biplot furnishes a comprehensive overview of genotype-environment (GxE) affinities, the stability of certain genotypes across diverse conditions, and the environmental distinctiveness that breeders must consider when targeting seed yield optimization and resilience.

**Figure 9:**
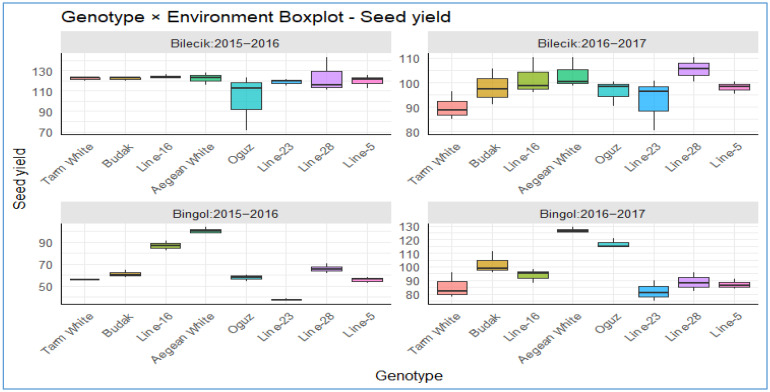
The distribution of seed yield across different genotypes in two distinct locations.

The box plots in Figure 9 offer a comprehensive comparative perspective on the seed yield performance (kg da^−1^) of eight genotypes: *Tarm White*, *Budak*, *Line-16*, *Aegean White*, *Oguz*, *Line-23*, *Line-28*, and *Line-5*. These genotypes were evaluated across two contrasting ecological locations (Bilecik and Bingol) over two consecutive growing seasons (2015–2016 and 2016–2017). In Bilecik’s temperate and relatively humid conditions, the overall yield levels were higher and more stable. All genotypes in Bilecik in the 2015–2016 growing season exhibited both elevated mean yields and diminished variance ranges, indicative of substantial resilience to climatic variations. In contrast, *Tarm White* and, to a lesser extent, *Budak* exhibited lesser yield ranges, particularly during the 2016–2017 season. All but *Line-5* in Bilecik in the 2016–2017 growing season show great variability, indicating heightened sensitivity to environmental stress. *Aegean White* and *Line-28* exhibited notable yield improvements in Bilecik during the 2016–2017 period, suggesting a positive adaptive response to seasonal conditions. In Bingol’s drier and harsher climate, average yields exhibited a pronounced decline and great yield variability across genotypes. However, certain genotypes, including *Aegean White*, *Line-16*, and *Oguz*, exhibited comparatively high mean yields and relatively narrow variance ranges. *Tarm White* maintained a balance of moderate-to-high yields with low variability across both locations, whereas *Line-5* consistently recorded lower yields across years and environments, hinting at possible genetic limitations. Collectively, these results offer critical insights into how genotypes respond to environmental conditions, providing valuable guidance for optimizing yield potential and developing strategies for climate resilience in modern agriculture.

Table 5 shows straw yields (kg ha^−1^) of Hungarian vetch genotypes, a key metric of biomass and adaptability. A comprehensive analysis of the data reveals significant disparities (*p* < 0.01) in yield outcomes across various years, geographical locations, and the interactions between location and year. This observation underscores the significance of genotype-by-environment (G × E) interactions, highlighting the impact of environmental factors on genotypic performance. Furthermore, the mean values across individual years were found to be statistically significant at the *p* < 0.05 level. This finding suggests the presence of temporal consistency. The *Aegean White* genotype exhibited the most substantial location-year interaction yield of 939.2 kg ha^−1^ at Bingol in 2015–2016, followed by *Line-28* at 919.1 kg ha^−1^ at the aforementioned site and year. In contrast, *Tarm White* exhibited the highest straw yield of 638.7 kg ha^−1^, while *Line-16* demonstrated the lowest yield of 443.1 kg ha^−1^ at Bilecik during the 2015–2016 period. Conversely, during the 2016–2017 growing season in Bilecik, *Budak* exhibited the highest straw yield, while *Line-16* demonstrated the lowest straw yield. The range of the two-year location interaction was from 492.7 to 663.3 kg per hectare, indicating genotypic variability. Consequently, *Aegean White* and *Line-28* emerge as promising candidates for inclusion in breeding programs aimed at enhancing Hungarian vetch production.

This information offers valuable insights for the development of targeted breeding and environmental matching strategies. In a study conducted by [28], the seed and straw yields of several Hungarian vetch lines and varieties cultivated under the arid conditions of Bingol were reported to range from 46.13 to 58.15 kg per day for seed yield and from 204.9 to 266.1 kg per day for straw yield. In a study by [42] that sought to ascertain the seed and straw yields, as well as the straw qualities, of certain Hungarian vetch genotypes, the range of seed yields was from 35.4 to 126.7 kg per day, while the range of straw yields was from 463.2 to 1110.0 kg per day. In contrast, [29] reported that the seed yields of certain Hungarian vetch varieties cultivated as bee pastures amounted to 22.5 kg per day, while [34] documented straw yields of 204 kg per day in a study aimed at ascertaining the straw yield and quality of select Hungarian vetch varieties.

**Table 5:** Straw yield (kg ha^−1^) of Hungarian vetch at both locations.

Genotypes	Locations						Year		Overall Mean
	Bilecik			Bingol			2015– 2016	2016– 2017	
	2015– 2016	2016– 2017	Mean Loc.	2015– 2016	2016– 2017	Mean Loc.			
*Tarm White*	638.7 a	537.0 cd	587.8 a	773.4 c	677.2 b	725.3 a	706.0 ab	607.1 a	656.6 a
*Budak*	595.4 ab	595.6 a	595.5 a	822.1 b	546.6 de	684.4 a	708.8 a	571.1 ab	639.9 a
*Line-16*	443.1 c	485.7 e	464.4 c	553.6 d	488.3 f	520.9 bc	498.4 d	487.0 c	492.7 c
*Aegean White*	555.1 b	560.6 bc	557.9 ab	939.2 a	589.4 cd	764.3 a	747.2 a	575.0 ab	661.1 a
*Oguz*	521.7 bc	507.7 de	514.7 b	587.6 d	723.9 a	655.8 ab	554.7 bcd	615.7 a	585.2 abc
*Line-23*	545.5 b	597.9 a	571.7 a	780.1 bc	470.0 f	625.0 abc	662.7 abc	533.9 bc	598.4 ab
*Line-28*	577.3 ab	561.6 bc	569.5 a	919.1 a	595.0 c	757.1 a	748.2 a	578.3 ab	663.3 a
*Line-5*	590.9 ab	591.8 ab	591.3 a	463.2 e	536.0 e	499.7 c	527.0 cd	563.9 ab	545.5 bc
**Overall mean**	558.5	554.7	556.6	7298	578.3	654.1	644.1	566.5	605.3
Aov (Gen.)	4.6 *** (*p* = 0.01)	15.6 *** (*p* = 0.00)	7.6 *** (*p* = 0.00)	121.2 *** (*p* = 0.00)	37.5 *** (*p* = 0.00)	3.6 *** (*p* = 0.00)			
Aov (Gen.xYear)	2.52 ** (*p* = 0.04)			78.82 *** (*p* = 0.00)					
**Statistical information about three way interaction:**									
Aov (Gen.xLoc.)	27.14 *** (*p* = 0.00)						26.5 *** (*p* = 0.00)	38.54 *** (*p* = 0.00)	2.91 *** (*p* = 0.00)
Aov (Gen.xYear)	26.24 *** (*p* = 0.00)								
Aov (Loc.xYear)	131.70 *** (*p* = 0.00)								
Aov (Gen.xLoc.xYear)	31.63 *** (*p* = 0.00)								

Note: **, and *** show statistical significance levels at 5%, and 1%, respectively. Also, means followed by different letters in the same column are significantly different according to the Least Significant Difference (LSD) test at *p* < 0.05 while the same letter indicates no statistically significant difference. Mean Loc. shows the mean locations while Aov stands for analysis of variance.

**Figure 10:**
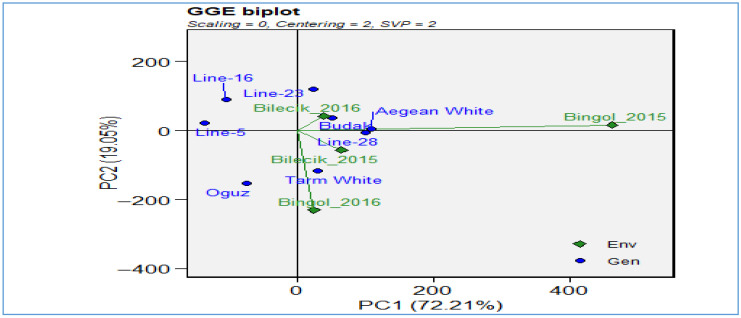
GGE biplot for straw yield.

Figure 10 demonstrates a robust assessment of genotypic performance and environmental effects. The first two principal components explain 91.26% of the total variation, affirming the biplot’s reliability. Bingol 2015 is positioned far to the right along PC1, indicating distinctly higher straw yield potential in this environment and a strong capacity to discriminate among genotypes. In contrast, Bilecik environments (2015 and 2016) exhibited a tendency to cluster near the origin, suggesting the presence of more moderate yields accompanied by relatively stable genotype responses. Conversely, Bingol 2016 is located in the lower quadrant, which may be indicative of more severe seasonal conditions. Genotypes such as *Aegean White*, *Budak*, and *Line-28*, located in proximity to the Bilecik vectors, appear to be better adapted to these environments, exhibiting a combination of moderate yields and stability. *Line-16* and *Line-5*, situated in the upper-left quadrant, manifested unique adaptation patterns; nevertheless, these patterns did not uniformly result in enhanced straw yield under the Bingol 2015 conditions. In contrast, *Oguz*, situated in the lower-left quadrant, exhibited an apparent lack of congruence with high-yielding environments such as Bingol 2015. The relative proximity of genotypes to environmental vectors underscores their suitability, with environmental dispersion highlighting inter-annual and locational differences. The biplot offers insights into which genotypes demonstrate optimal performance under specific conditions and which environments are most conducive to identifying superior straw-yield performance.

**Figure 11:**
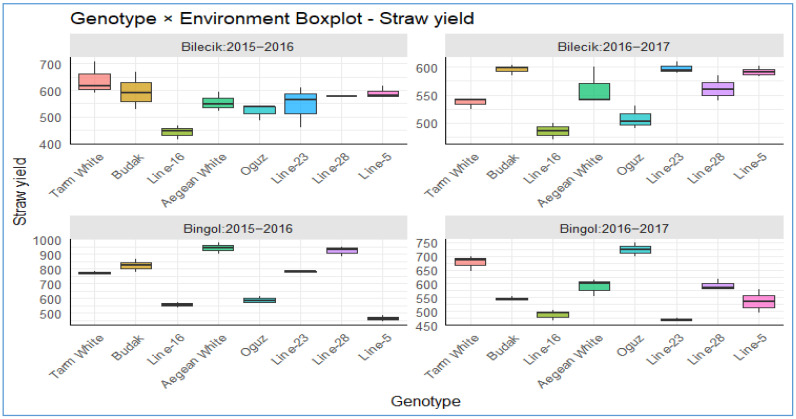
The distribution of straw yield across different genotypes in two distinct locations.

The boxplots depicted in Figure 11 offer a compelling comparative analysis of straw yield distributions (kg da^−1^) for a range of genotypes grown under Bilecik’s temperate, humid climate and Bingol’s drier, more variable conditions over the 2015–2016 and 2016–2017 seasons. This analysis elucidates the pivotal role of genotype–environment interactions in shaping agricultural adaptation and yield stability within the context of contemporary agronomy. In Bilecik, genotypes demonstrated consistent and high performance within the 450–650 kg da^−1^ range. It is noteworthy that low-variance lines, exemplified by *Tarm White*, *Line-23*, and *Line-28*, with their narrow box ranges, exhibited remarkable resilience to climatic variations. These lines consistently yielded stable harvests of approximately over 600 kg per day, exhibiting only a marginal inter-seasonal decline from 2015 to 2016 to 2016 to 2017. *Budak* and *Line-23* exhibited moderate yields and low variability at Bilecik in 2016–2017 period, indicative of their inherent genetic potential. In contrast, genotypes such as *Aegean White*, *Oguz*, and *Line-28* exhibited broad box widths, indicating substantial variability and yield losses, particularly during the 2016–2017 period. These findings suggest that they exhibit a degree of vulnerability to drought-like stresses. In contrast, the more austere climate experienced in Bingol resulted in a significant varying in yields, with yields ranging from 450 to 950 kg. In this instance, the persistently low-performing *Line-5*, *Line-16*, and *Line-23* (narrow boxes yet below 500 kg da^−1^) highlighted genetic constraints and suboptimal climatic suitability, while *Aegean White* and *Oguz* once again exhibited comparatively high yields with minimal variability, manifesting indications of inter-seasonal enhancement and extensive adaptation potential, compatible with above findings. Conversely, *Line-23* exhibited substantial variability and a pronounced decrease in yield during the 2016–2017 period. This decline is substantial. Taken together, these findings underscore—from an inspiring, breeding-oriented perspective—that in the era of climate change, the selection of resilient genotypes, exemplified by stable performers, is imperative.

The harvest index (HI) of Hungarian vetch genotypes varied significantly across locations and years (Table 6). Among the tested genotypes, *Line-16* consistently recorded the highest HI, with a two-year overall mean of 21.0%, indicating superior efficiency in partitioning HI. *Aegean White* also performed well (18.2%), whereas *Line-23* (14.9%) and *Tarm White* (13.9%) exhibited the lowest values. Location means revealed that Bilecik (13.2%) supported a higher HI than Bingol (9.4%), suggesting more favorable environmental conditions for reproductive allocation in Bilecik. Across years, HI was higher in 2016–2017 (17.5%) than in 2015–2016 (15.5%), likely reflecting improved climatic conditions in the second year.

Significant genotype × location and genotype × year interactions, along with a highly significant genotype × location × year interaction, highlight the influence of both environmental variability and genetic potential on HI expression. These results are in line with previous findings that HI is a genotype-dependent trait strongly modulated by environmental conditions [28,29,30,31,32,33]. Therefore, breeding programs aiming to improve Hungarian vetch for seed productivity should consider selecting genotypes like *Line-16*, which combine high yield potential with stability across contrasting environments.

**Table 6:** Harvest index (%) of Hungarian vetch at both locations.

Genotypes	Locations						Year		Overall Mean
	Bilecik			Bingol			2015– 2016	2016– 2017	
	2015– 2016	2016– 2017	Mean Loc.	2015– 2016	2016– 2017	Mean Loc.			
*Tarm White*	19.2 b	16.8 bc	17.9 b	7.2 d	12.7 e	9.9 c	13.2 b	14.7 c	13.9 c
*Budak*	20.7 b	16.5 bc	18.6 b	7.5 d	18.7 bc	13.1 abc	14.1 ab	17.6 b	15.8 bc
*Line-16*	28.1 a	21.0 a	24.5 a	15.8 a	19.2 ab	17.5 a	21.9 a	20.1 a	21.0 a
*Aegean White*	22.1 b	18.5 ab	20.3 b	10.8 c	21.5 a	16.1 ab	16.4 ab	20.0 a	18.2 ab
*Oguz*	19.7 b	19.0 ab	19.4 b	9.8 c	16.2 cd	12.9 abc	14.8 ab	17.6 b	16.2 bc
*Line-23*	22.1 b	15.4 c	18.8 b	4.8 e	17.5 bc	11.1 bc	13.5 b	16.5 bc	14.9 bc
*Line-28*	21.4 b	18.8 ab	20.1 b	7.2 d	14.9 de	11.0 bc	14.3 ab	16.9 bc	15.6 bc
*Line-5*	20.3 b	16.6 bc	18.4 b	12.1 b	16.3 cd	14.2 abc	16.2 ab	16.4 bc	16.3 bc
**Overall mean**	21.7	17.8	19.8	9.4	17.1	13.2	15.5	17.5	16.5
Aov (Gen.)	2.5 * (*p* = 0.06)	4.1 *** (*p* = 0.01)	2.5 ** (*p* = 0.03)	71.9 *** (*p* = 0.00)	10.7 *** (*p* = 0.00)	1.9 (*p* = 0.10)			
Aov (Gen.xYear)	1.18 (*p* = 0.34)			13.69 *** (*p* = 0.00)					
**Statistical information about three way interaction:**									
Aov (Gen.xLoc.)	2.64 ** (*p* = 0.02)						2.31 * (*p* = 0.05)	5.51 *** (*p* = 0.00)	0.58 (*p* = 0.77)
Aov (Gen.xYear)	2.94 *** (*p* = 0.00)								
Aov (Loc.xYear)	225.60 *** (*p* = 0.00)								
Aov (Gen.xLoc.xYear)	3.99 *** (*p* = 0.00)								

Note: *, **, and *** show statistical significance levels at 10%, 5%, and 1%, respectively. Also, means followed by different letters in the same column are significantly different according to the Least Significant Difference (LSD) test at *p* < 0.05 while the same letter indicates no statistically significant difference. Mean Loc. shows the mean locations while Aov stands for analysis of variance.

**Figure 12:**
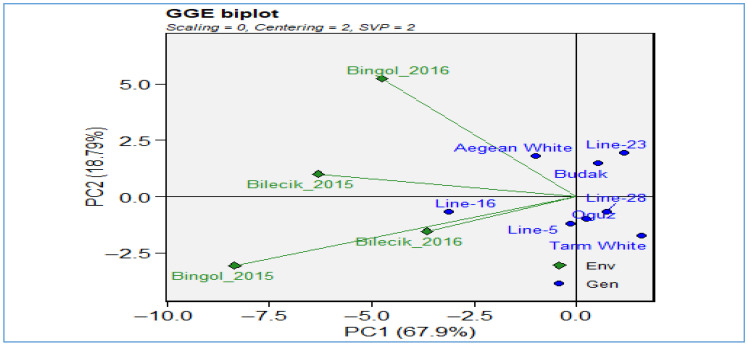
GGE biplot for harvest index.

Figure 12 of the GGE biplot analysis provides an in-depth examination of genotype-by-environment interactions for the harvest index trait, explaining 86.7% of the total variation (PC1: 67.9%, PC2: 18.8%) and thus offering a highly reliable representation. The biplot provides a visual representation of the environments of Bilecik 2015–2016 and Bingol 2015–2016 (depicted as green diamond markers) alongside the genotypes *Line-16*, *Line-23*, *Line-28*, *Line-5*, *Aegean White*, *Budak*, *Oguz*, and *Tarm White* (depicted as blue dot markers). This enables a comparative assessment of their performance. The spatial configuration of the environments in question reveals that both locations are positioned along the negative PC2 axis. This highlights fundamental similarities in harvest index between the two regions and underscores the substantial influence of environmental factors, such as climate, soil characteristics, and management practices, on genotype performance. With respect to genotype distribution, *Line-23*, *Line-28*, *Budak*, *Oguz* located at positive PC1 values, demonstrate superior adaptation to the Bingol environment, while *Line-16* and several other genotypes cluster near the origin, reflecting broad adaptation potential. In contrast, Aegean White and *Tarm White* display a distinct ecological niche, suggesting a unique environmental predilection. The biplot under consideration provides quantitative evidence of the magnitude of the genotype-environment (G × E) interaction for the harvest index. Furthermore, it emphasizes the significance of location-specific selection strategies within breeding programs. Furthermore, it offers insights into the discriminative ability and representativeness of environments, which contributes to the identification of breeding targets within the framework of the ideal genotype and ideal environment concepts.

**Figure 13:**
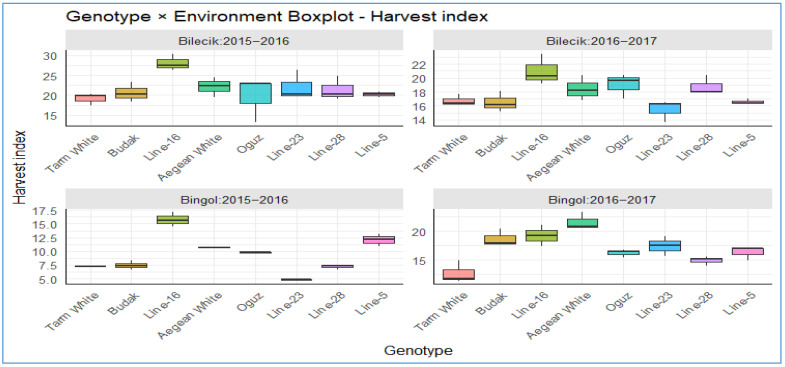
The distribution of harvest index across different genotypes in two distinct locations.

Figure 13 delivers a comprehensive biplot analysis of harvest index distribution across different growing seasons and areas. In Bilecik, during both years, *Line-16* and *Aegean White* show higher harvest indices, peaking around 20–25, while *Line-5* and *Line-23* are lower at 15–20. In 2016–2017, *Oguz* and *Line-23* lead with indices around 18–22, whereas *Tarm White* and *Budak* drop to 14–18. In Bingol, during 2015–2016, genotypes like *Line-16* peak the highest value, while *Aegean White* genotype hits the highest value in 2016–2017 season. Also, *Tarm White*, *Budak*, and *Aegean White*, *Oguz*, and *Line-5* achieve indices up to 14–15 in 2015–2016 period, while in 2016–2017 period, *Budak*, *Line-16*, and *Aegean White* reach 15–22, while *Oguz*, *Line-23*, *Line-28*, and *Line-5* remains lowest at 15–17%. Overall, Bilecik supports higher average harvest indices (15–30%) compared to Bingol (7–22), indicating that environmental factors significantly influence genotype performance.

## 4. Conclusions

The present study examined seed yield and its components in Hungarian vetch genotypes across two contrasting environments, namely Bilecik and Bingol. The GGE Biplot analysis was utilized in order to elucidate genotype–environment interactions. The performance of the genotype exhibited significant variation between the two locations, contingent upon the specific trait under consideration. For certain key traits, some genotypes achieved remarkably elevated yields, whereas others exhibited a combination of enhanced environmental resilience and comparatively diminished yield levels.

In general, under Bilecik conditions, the genotypes *Tarm White*, *Aegean White*, *Oguz*, and *Line-23* exhibited higher yield levels for the primary traits evaluated. In Bingol, *Aegean White* and *Oguz* were the leading performers in terms of yield, while *Line-5* was notable for its resilience. Furthermore, GGE Biplot and principal component analyses indicated that in Bilecik, the genotypes *Oguz*, *Line-16*, and *Line-5* were notable for their harvest index, number of pods per plant, and seed yield, whereas in Bingol, *Aegean White* and *Line-23* were more prominent. It is noteworthy that the three line-type genotypes—*Line-5*, *Line-23*, and *Line-28*—exhibited low variability and high environmental stability, indicative of minimal sensitivity to environmental fluctuations.

The findings emphasize the pivotal role of genotype-environment interactions in shaping yield components and underscore the efficacy of GGE Biplot analysis in identifying high-performing cultivars adapted to specific regions. In particular, genotypes such as *Aegean White*, *Oguz*, and *Line-16* exhibited a combination of high yield potential and environmental stability, rendering them valuable candidates for sustainable forage production under Türkiye’s variable climatic conditions. The insights derived from this study can inform region-specific genotype selection, thereby enhancing productivity and improving resource-use efficiency.

A potential limitation of the present study is that other environmental variables, such as temperature, precipitation, and humidity, were assumed to be homogeneous. Future research could refine genotype–environment interaction (GEI) analyses by incorporating variation in these factors, enabling a deeper understanding of their influence on genotype performance.

## Data Availability

The dataset generated and analyzed during this study is available in the Bona Res Data Centre repository or upon reasonable request from the corresponding author.
